# Elevated zinc concentrations in a 5 months old infant: A case report

**DOI:** 10.11613/BM.2018.011001

**Published:** 2018-01-10

**Authors:** Eva Rabing Brix Petersen, Sven Mortensen, Mads Nybo

**Affiliations:** 1 Department of Clinical Biochemistry and Pharmacology, Odense University Hospital, Odense, Denmark; 2 Department of Pediatrics, Odense University Hospital, Odense, Denmark

**Keywords:** capillary blood sample, case report, pre-analytical phase, zinc

## Abstract

Pre-analytical errors account for the majority of laboratory-associated errors. In a 5 months old infant hospitalised with lung dysfunction due to prematurity, a routine measurement of zinc revealed an unexpected elevated concentration of 20.2 µmol/L (reference interval 10.0 - 19.0 µmol/L) compared to 11.6 µmol/L five days earlier. Zinc measurement was repeated two days later and had further increased to 42.4 µmol/L. Of note, there were no clinical signs of the increased zinc concentrations. Performance data for the zinc analysis (performed by inductively coupled plasma mass spectrometry) was found satisfactory. A thorough review of the patient´s medication and nutrition supplements revealed no relevant zinc content. The blood was obtained through capillary blood sampling, and anything at the skin puncture site containing zinc could therefore potentially contribute to the elevated zinc results. It was investigated if any ointment containing zinc had been applied at the puncture site, which revealed that the mother had applied vitamin E ointment containing zinc-oxide at the infant’s heel. A capillary sample obtained from the opposite heel, where no vitamin E ointment had been applied, revealed a zinc concentration of 14.3 µmol/L. In conclusion, pre-analytical contamination with ointments must be considered in case of unexpected measurements from capillary blood.

## Introduction

Pre-analytical errors account for the majority of laboratory errors and are seldom assessed and subsequently remain unaddressed (*1*, *2*). When unexpected test results emerge one must always think of the path of the sample. This especially goes for children, where blood sampling often is performed in an alternate way, *e.g.* through catheters, as capillary sampling or with too little blood material. We here report a case of unexpected elevated zinc concentrations in a 5 months old infant. Informed consent was obtained from the mother of the child and is correctly stated in the patient’s records.

## Laboratory analyses and case story

Department of Clinical Biochemistry was contacted by a paediatrician who had noticed a slight increase in zinc concentrations in a 5 months old infant hospitalised with lung dysfunction due to prematurity: the zinc concentration was 20.2 µmol/L (reference interval 10.0 - 19.0 µmol/L) compared to 11.6 µmol/L five days earlier. As this finding was unexpected, the analysis was repeated after two days only to find a further increase in zinc concentration, namely 42.4 µmol/L (Table 1). The paediatrician relevantly posed the question if it could be due to an erroneous measurement or interference of the analysis as the increase in zinc concentration was deemed unlikely. As far as the paediatrician knew, the infant did not receive any zinc supplements. Of note, the increase in zinc concentration occurred during the infant´s stay at the paediatric intensive care unit, whereas the normal values were obtained during the stay at the Department of Pediatrics. In order to unravel this, a number of focus areas were investigated.

**Table 1:** Laboratory results for zinc concentration

**Parameter**	**Time of sample**	**Value**	**Reference interval**
Zinc (µmol/L)	Day 1	11.6	10.0 - 19.0
	Day 6	20.2	
	Day 8	42.4	
	Day 11	14.3	

### Erroneous substitution

A thorough review of the manufacturers’ summary of product characteristics for the medication and nutrition supplements (ABIDEC multivitamins, D-vitamins, Movicol, Sildenafil, Diural and Budesonid) revealed no relevant content of zinc. The laboratory offered to determine the exact content of zinc in the different formulas if the zinc concentration by mistake should be increased in any of the supplements or medication given despite the declarations of content, which was not found necessary by the paediatricians. The latter suggestion was based on a recent finding of severely increased vitamin D concentrations in oral supplementations, which led to vitamin D intoxications and the withdrawal of this particular product (*3*).

### The analysis

The zinc analysis is performed using inductively coupled plasma mass spectrometry (iCAP-Qc ICP-MS, Thermo Fisher, Winsford, UK), and the internal and external controls were inspected and found satisfactory. Even though mass spectrometry is a robust method, haemolysis is recognised as a potential source of interference on zinc measurement (*4*). In this case there was no suspicion of haemolysis, and it was also assured that the sample had been drawn in the tube intended for zinc analysis (BD Vacutainer trace element tube ref. 368380, Lyngby, Denmark). The laboratory technician responsible for the trace element laboratory actually remembered this particular sample as zinc measurements are rarely elevated, and on her initiative the sample had already been re-analysed to ensure a correct result.

### The sampling

The samples used for the zinc analyses were capillary blood samples (Tenderfoot Newborn Heel Incision Device, Accriva Diagnostics, San Diego, USA). Capillary sampling is in many aspects more advantageous than venous blood sampling as it is less invasive, often easier to carry out and demands less amounts of blood for the analyses, which especially in infants plays an important role in avoiding iatrogenic anaemia (*5*, *6*). The drawback is that capillary sampling is more error prone and if carried out erroneously it can lead to inaccurate test results (*5*, *7*-*9*).

The phlebotomists working at the paediatric intensive care unit are specially trained to obtain paediatric samples, but the procedure for capillary blood sampling is the same at the paediatric intensive care unit and at the department of Pediatrics. It therefore seemed unlikely that the blood sampling in itself should have contributed to the discordant results as the techniques used are the same at the paediatric intensive care unit and at the department of Pediatrics. The specially educated paediatric phlebotomists were re-informed on the necessity of performing as atraumatic sample retrieval as possible. Notable, it was confirmed by the phlebotomists that no ointment containing zinc was applied at the puncture site. To assure this the Department of Clinical Biochemistry did however contact the paediatricians and encouraged them to investigate whether anything with zinc content had been used on the infants skin from where the capillary blood was drawn.

## Solution

An hour later the paediatrician could inform that the mother, in order to improve skin healing, had applied vitamin E ointment containing zinc-oxide at the infant’s heel, where the numerous capillary samplings were performed. A capillary sample was obtained three days later from the opposite heel, where no vitamin E ointment containing zinc-oxide had been applied, and this confirmatory test revealed a zinc concentration of 14.3 µmol/L (*i.e.* within the reference interval). It could therefore be concluded that it was the pre-analytical contamination with zinc-oxide in the vitamin E ointment, which had led to the elevated zinc concentrations.

## Discussion

In case of unexplained or unexpected analyses results or in cases where the analyses results do not match the clinical symptoms, pre-analytical errors must always be considered and addressed systematically, including how the sample material was obtained. It is important to standardise sampling techniques, *e.g.* following the Clinical and Laboratory Standards Institute (CLSI) Guideline for capillary sampling (*6*). It is however important to be aware of possible pre-analytical reasons for unexpected test results and in order to solve these problems the laboratory professionals must be keen on following the pre-analytical path of the sample.

As depicted here, a structured approach starting with the analysis, investigating other sources of the element at quest (here zinc) and then of course following the sample from puncture to laboratory, are all vital steps that must be scrutinised. Under such circumstances, a close contact and dialogue between the clinician and the clinical biochemist is of utmost importance, as the chances of finding the correct – often pre-analytical – cause thereby are increased considerably.

The initial zinc measurement was part of a standard profile at the paediatric intensive care unit and there was no specific indication for the measurement. It is well-known that multiple testing can cause harm to the patient and - as illustrated in this case - it can lead to unnecessary anxiety for both the patients and the professionals. In order to reduce the iatrogenic effects of multiple unjustified testing the clinicians should always have a clear indication for test ordering. There are many approaches towards better test management, amongst others the Choosing wisely initiative, which intends to reduce waste in health care and avoid unnecessary tests and invasive procedures and consequently decrease patient risk and costs (*10*).

For future studies as a final prove that it was the pre-analytical contamination with zinc-oxide in the vitamin E ointment, which had led to the elevated zinc concentrations, we recommend testing at volunteers to investigate if the administration of the ointment influences zinc measurement.

## What YOU should / can do in your laboratory to prevent such errors

Altogether, the following learning points can be extracted from the presented case:

When something seems wrong, it often is wrong.
For children: Ask the parents!
Capillary sampling: As the blood is obtained through skin puncture instead of venepuncture, it is even more important to address the actual sampling procedure thoroughly.
Be aware that over-testing can lead to overtreatment and endanger the patient.
